# Oculomotor nerve palsy associated with bortezomib in a patient with multiple myeloma: a case report

**DOI:** 10.1186/1752-1947-4-342

**Published:** 2010-10-26

**Authors:** Bassem Toema, Hamdan El-Sweilmeen, Tarek Helmy

**Affiliations:** 1 Division of Hematology and Oncology, Internal Medicine Department, Saad Specialist Hospital, Prince Faisal Bin Fahed Street, P.O. Box 30353, AlKhobar, 31952, Saudi Arabia; 2 Radiology Department, Saad Specialist Hospital, Prince Faisal Bin Fahed Street, P.O. Box 30353, AlKhobar, 31952, Saudi Arabia

## Abstract

**Introduction:**

Bortezomib is a proteasome inhibitor used in the treatment of multiple myeloma. A newly recognized oculomotor nerve palsy related to bortezomib is described.

**Case presentation:**

A 54-year-old Caucasian woman with immunoglobulin G kappa multiple myeloma on single-agent bortezomib given by intravenous push once weekly developed isolated unilateral partially reversible left sided oculomotor nerve palsy during the first cycle of treatment. All the essential diagnostic tests that were carried out excluded all other possible causes. There was a positive dechallenge-rechallenge test. Management was by withdrawal of bortezomib and empirical dexamethazone. To the best of our knowledge, this is the first report of its kind in the literature.

**Conclusion:**

This case illustrates the probable association between oculomotor nerve palsy and bortezomib, and generates a hypothesis of whether bortezomib can cross the blood-brain barrier or not.

## Introduction

Bortezomib is a 26S proteasome inhibitor which activates signaling cascades, cell cycle arrest and apoptosis. Intravenous bortezomib is a recommended treatment in multiple myeloma, as demonstrated in the phase II CREST and SUMMIT trials, and the phase III APEX trial. Most of the reports regarding neurologic adverse events of bortezomib relate to associated peripheral neuropathy. None reported associated cranial neuropathies. We are reporting this adverse event to describe a newly recognized possible adverse reaction or interaction related to bortezomib which is oculomotor nerve palsy. To the best of our knowledge, this is the first report of this kind in the literature.

## Case presentation

A 54-year-old Caucasian woman had a positive family history for hypertension and negative family history for malignancy, with hypertension controlled by enalapril and atenolol and open angle glaucoma controlled by latanoprost eye drops. She was diagnosed with immunoglobulin G kappa multiple myeloma and started bortezomib as a first line therapy for multiple myeloma. She received bortezomib as a single agent (1.3 mg/m^2^; total dose of 2 mg) via intravenous push once weekly for multiple myeloma. The treatment regimen was given in a non standard way without concomitant dexamethazone. She received Cycle 1 Day one, Cycle 1 Day eight, Cycle 1 Day 15 and developed isolated unilateral partially reversible left sided oculomotor nerve palsy on Cycle 1 Day 21.

The developed isolated unilateral partially reversible left sided oculomotor nerve palsy was graded as II according to National Cancer Institute's Common Toxicity Criteria Version 2.0, as there was partial weakness of levator palpebrae muscle power resulting in mild partial ptosis of the left eye and persistent impairment of the third nerve mediated extraocular muscle movement. This resulted in complete loss of medial movement 'adduction' of left eye, divergent squint and partially defective upward 'elevation' and downward 'depression' movement of the left eye. The objective weakness is mild, interfering with function, but not interfering with activities of daily living.

Management of this adverse drug event was by withdrawal of the drug bortezomib by omitting Cycle 1 week four (Day 22) of bortezomib and replacing it with an intravenous infusion of dexamethazone (8 mg) once daily for four days on Cycle 1 Day 22, Cycle 1 Day 23, Cycle 1 Day 24 and Cycle 1 Day 25. On Cycle 1 Day 27 good partial improvement of the oculomotor nerve palsy was noted and therefore Cycle 2 Day one of bortezomib was given. On Cycle 2 Day three there was strong reappearance of most of the signs of left sided oculomotor nerve palsy and despite reintroducing a dexamethazone 8 mg intravenous infusion once daily for four days on Cycle 2 Day four, Cycle 2 Day five, Cycle 2 Day six and Cycle 2 Day seven to ameliorate the signs of oculomotor nerve palsy, yet the response noted was not as striking as before and the improvement was nil leaving our patient with residual oculomotor nerve palsy. Eventually bortezomib was discontinued and our patient shifted to melphalan-lenalidomide combination therapy.

## Discussion

The case presented here showed suggestive evidence linking the drug to the event. To associate bortezomib to the oculomotor nerve palsy, we had to rule out all other possible causes, assess the temporal relationship and pharmacological time plausibility, and confirm positive dechallenge/rechallenge response.

Our patient's only known comorbidities are hypertension of ten years duration controlled by enalapril and atenolol, and open angle glaucoma of two months duration controlled by latanoprost eye drops. These three medications (enalapril tablets, atenolol tablets and latanoprost eye drops) are not reported to cause oculomotor nerve palsy or any other similar cranial nerve palsy or neuropathy. Furthermore, she had used enalapril tablets and atenolol tablets for ten years and latanoprost eye drops for two months without developing this adverse event. She has no past history of any cerebrovascular accident or any thromboembolic event. She is not known to be diabetic and not known to suffer from peripheral vascular disease. She never complained of any similar incident of cranial nerve palsy or even peripheral neuropathy.

Fundoscopy was carried out and it showed the optic disc to be within normal appearance, no papilloedema was detected. A beta scan of both eyes was done and showed bilateral normal retinochoroidal thickness and bilateral normal optic nerve thickness.

Magnetic resonance imaging (MRI) of the brain and brain stem with and without contrast was carried out and the only finding was a focal enhancing area in the white matter of the right pons about 11 mm in maximum diameter with low T1 and high T2 signal. This area showed diffuse enhancement in the post contrast study and there is no surrounding edema detected. This was graded as a non-specific appearance and differential diagnosis includes demyelination and minute cerebrovascular accident. A decision was taken not to follow this non-specific lesion by a repeat MRI of the brain and brain stem since this lesion is right sided and usually left oculomotor nerve palsy is expected to occur by an ipsilateral structural lesion on the same side.

Cerebrospinal fluid (CSF) was obtained by lumbar puncture. Cytological examination of the CSF sample was negative for malignant cells. An analysis and cell count of the CSF sample showed negative criteria for subarachnoid hemorrhage, multiple sclerosis and/or viral and bacterial meningitis. Glucose level in the CSF sample was 4.4 mmol/L (reference range: 2.2 to 3.9), the protein level in the CSF sample was 440 mg/L (reference range: 120 to 600). The CSF sample total volume was 1.3 mL, with clear appearance and was colorless, white blood cell count in the sample was three cells per microliter (reference range: zero to five) and red blood cell count in the sample was zero cells per microliter (reference range: zero to five). CSF culture and sensitivity showed no growth after 72 hours of incubation and no growth after enrichment culture.

Nerve conduction studies were not done because although our patient complained of numbness and peripheral paraesthesia, they were mild and did not interfere with the activities of daily living.

Despite the CSF cytology being negative for malignant cells and a MRI of the brain being inconclusive, it is impossible to rule out with 100% certainty extramedullary myelomatous infiltration of the brain. The only point that suggests that this adverse event wasn't related to multiple myeloma was that the serial serum IgG level which was used as a biomarker to follow the status of her disease was dropping from baseline of 53.4 g/L before initiation of the bortezomib to 23.5 g/L on the day she developed this adverse event meaning that her disease was responding to bortezomib.

This is a plausible collateral adverse event that occurred early as regards time onset. Our patient was on bortezomib (1.3 mg/m^2^; total dose of 2 mg) as a first line monotherapy that was administered as intravenous push once weekly, she received Cycle 1 week 1 (Day one), Cycle 1 week two (Day eight), Cycle 1 week three (Day 15) and developed the adverse event on Cycle 1 Day 21. According to the DoTS classification, this is probably a collateral effect of early persistent or intermediate time-course; the susceptibility factors are not known [1].

To the best of our knowledge, there has been no previously reported or published recognized association (oculomotor nerve palsy) or even similar association (cranial neuropathy) with the product 'bortezomib' or the class 'proteasome inhibitor'. There have been several reports of peripheral neuropathies with the product 'bortezomib' or the class 'proteasome inhibitor'.

Plasma level of bortezomib or its metabolite was not assessed at the time of the adverse event.

There is insufficient animal and *in vitro *data regarding the association of cranial nerve palsy with bortezomib as a possible adverse event related to the drug.

Omitting Cycle 1 week four (Day 22) of bortezomib resulted in partial improvement of the signs of oculomotor nerve palsy. Partial improvement was noticed on Cycle 1 Day 27 i.e. after five days of omitting the dose of bortezomib.

Giving cycle 2 week one (Day one) of bortezomib resulted in reappearance of most of the signs of left sided oculomotor nerve palsy after 48 to 72 hours of reintroducing bortezomib.

## Conclusion

This is a case report of a single patient. The drug implicated is bortezomib. There is probably a true association linking oculomotor nerve palsy to bortezomib (score of 7 according to Naranjo algorithm). Some suggested reasons are the temporal relationship and pharmacological time plausibility, positive dechallenge/rechallenge and all other possible causes for oculomotor nerve palsy were ruled out.

The hypothesis generated is: does bortezomib cross the blood-brain barrier or not and can bortezomib cause cranial neuropathy or not? There are no relevant published pharmacokinetic studies regarding the ability of bortezomib to cross the blood-brain barrier. Further observational studies are warranted.

The mechanism for this adverse drug event is not known. It is proposed to be either direct neurotoxicity of bortezomib or modulation of the inflammatory and immune responses via affecting function and survival of immune cells such as lymphocytes and dendritic cells [2]. The effect of bortezomib may be similar to that of immunosuppressive or immunomodulating agents, such as cyclosporin, tumor necrosis factor alpha antagonists (infliximab, etanercept, adalimumab), or to that of autologous peripheral blood stem cell transplantation, which have all been reported to precipitate both acute and chronic inflammatory demyelinating neuropathies [3-6]. In case of the associated neuropathy of tumor necrosis factor alpha antagonists, the possible mechanisms of action include both T-cell and humoral immune attack against peripheral nerve myelin, vasculitis-induced nerve ischemia, and inhibition of signaling support for axons [7].

The implications for clinical practice include that patients should undergo a standard neurological examination before starting bortezomib, and close clinical follow-up should be assured to reduce dosage or discontinue bortezomib in the case of the appearance, persistence or worsening of neurological symptoms. Should the neurological impairment worsen despite bortezomib dose reduction or discontinuation, the administration of an immune treatment such as steroids or intravenous immunoglobulins may be considered, assuming that most likely underlying mechanism is immune-mediated neuropathy [8].

## Consent

Written informed consent was obtained from the patient for publication of this case report and any accompanying images. A copy of the written consent is available for review by the Editor-in-Chief of this journal.

## Competing interests

The authors declare that they have no competing interests.

## Authors' contributions

HE analyzed and interpreted the patient data regarding multiple myeloma and was a major contributor in writing the manuscript. TH performed the magnetic resonance imaging of the brain and interpreted the data. All authors read and approved the final manuscript.

## References

[B1] Aronson JK Ferner RE Joining the DoTS New approach to classifying adverse drug reactions BMJ 2003 327 1222 1225 10.1136/bmj.327.7425.1222 14630763 PMC274067

[B2] Nencioni A Grunebach F Patrone F Ballestrero A Brossart P The Proteasome and its inhibitors in immune regulation and immune disorders Journal of Critical Revision of Immunology 2006 26 6 487 498 10.1615/critrevimmunol.v26.i6.20 17341190

[B3] Peters G Larner AJ Chronic inflammatory demyelinating polyneuropathy after autologous peripheral blood stem cell transplantation Journal of Peripheral Nervous System 2005 10 4 384 385 10.1111/j.1085-9489.2005.00052.x 16279989

[B4] Richez C Blanco P Lagueny A Schaeverbeke T Dehais J Neuropathy resembling Chronic Inflammatory Demyelinating Polyneuropathy in patients receiving tumor necrosis factor-alpha blockers Journal of Neurology 2005 64 8 1468 1470 10.1212/01.WNL.0000158681.29117.8B 15851749

[B5] Shin IS Baer AN Kwon HJ Papadopoulos EJ Siegel JN Guillain-Barré and Miller Fisher syndromes occurring with tumor necrosis factor alpha antagonist therapy Journal of Arthritis Rheumatology 2006 54 5 1429 1434 10.1002/art.21814 16645971

[B6] Terenghi F Ardolino G Nobile-Orazio E Guillain-Barré syndrome after combined CHOP and rituximab therapy in non-Hodgkin lymphoma Journal of Peripheral Nervous System 2007 12 2 142 143 10.1111/j.1529-8027.2007.00134.x 17565540

[B7] Stubgen JP Tumor necrosis factor-alpha antagonists and neuropathy Journal of Muscle Nerve 2008 37 3 281 292 10.1002/mus.20924 18041052

[B8] Ravaglia S Corso A Piccolo G Lozza A Alfonsi E Mangiacavalli S Varettoni M Zappasodi P Moglia A Lazzarino M Costa A Immune-mediated neuropathies in myeloma patients treated with Bortezomib Journal of Clinical Neurophysiology 2008 119 2507 2512 10.1016/j.clinph.2008.08.007 18829381

